# Re-Audit of the Monitoring of National Early Warning Scores 2 (NEWS2) in Old Age Patients

**DOI:** 10.1192/bjo.2026.11688

**Published:** 2026-06-30

**Authors:** Sadia Tabassum Javaid, Bethany Brace, Jeremy Virr, Jacob Baskeyfield, Sumara Nawaz

**Affiliations:** North Staffs Combined Healthcare NHS Trust, Stoke-On-Trent, United Kingdom

## Abstract

**Aims:**

The NEWS2 tool is integral to post-admission physical health monitoring, guiding baseline assessment and early detection of deterioration. It standardises observation recording and escalation. Trust Standard Operating Procedure and the Physical Health Policy provide detailed guidance for assessment, recording, and escalation, in alignment with NICE standards. Following an initial audit identifying documentation and escalation gaps, recommendations were implemented, including standardisation and increased electronic recording. This re-audit assesses the impact of these interventions.

**Aim and Objectives**

- To compare re-audit results with the previous audit to assess progress against Trust standards.
- To evaluate the impact of initial audit recommendations on correct NEWS2 utilisation.
- To identify persistent issues in NEWS2 monitoring or recording and inform further action.
- To enhance patient care and safety.

**Methods:**

We conducted a comprehensive review of each section of NEWS2 charts using the same proforma as the initial audit for 35 patients admitted to wards 6 and 7 at Harplands Hospital over three weeks. Patients were aged 68–91 years with near-equal gender distribution, no pregnancies. Findings were analysed using SPSS and compared to both Trust standards and previous audit results to assess improvements in NEWS2 monitoring and recording.

**Results:**

In the re-audit, NEWS2 charts were completed on admission for 33/35 patients, compared with 37/39 previously. Electronic recording predominated, with all 35 patients having electronic charts, 12 of whom also had paper charts; previously, electronic charts were often left unfilled.

Documentation quality improved markedly: patient demographics, date, time, and assessor name were completed in 100% of charts. Previously, 12 charts were incomplete, 9 had illegible entries, 6 missing signatures,12 incomplete sections, 9 lacked demographics, 13 had date/time missing. All physiological observations, including blood glucose, were accurately recorded, paper chart dots joined, and NEWS scores correctly calculated for all patients. Two patients scored ≥3 on ACVPU, with appropriate Glasgow Coma Scale documentation, compared to incomplete GCS recordings in two charts previously.

Weekly NEWS monitoring occurred in 31/35 patients; the remainder had documented clinical justification. Escalation improved: NEWS scores 1–4 were reviewed by nursing staff in 20/21 escalated cases (previously inconsistently reviewed), and all scores >4 were escalated to medical staff, with emergency services contacted if medics were unavailable.

**Conclusion:**

Implementation of previous recommendations improved consistent and correct NEWS2 use, supported by electronic documentation. Appropriate escalation of clinically significant scores strengthened patient safety. Performance improved across all domains, meeting Trust and national standards. Findings have been disseminated, with ongoing efforts to sustain performance.

